# Supplementary material for: Night-time hot spring bathing is associated with improved blood pressure control: A mobile application and paper questionnaire study
Source: PLoS One. 2024 Nov 1;19(11):e0299023. doi: 10.1371/journal.pone.0299023 (PMC11530088; doi:10.1371/journal.pone.0299023)
Supplement: S1 Table — (DOCX) [file pone.0299023.s002.docx]

**Questionnaire on night-time hot spring bathing and blood pressure**

Q1. Gender

1. Male 2. Female

Q2. Age

_______ years old

Q3. Disease history

Please check the relevant box(es) to indicate which (if any) disease(s) you have experienced.

□① Hypertension (with medication)

□② Hypertension (without medication)

□③ Cancer

□④ Acute myocardial infarction and angina

□⑤ Arrythmia

□⑥ Stroke

□⑦ Gout

□⑧ Diabetes mellites

□⑨ Hyperlipidemia

□⑩ Renal disease

□⑪ Depression

□⑫ Collagen disease (e.g., rheumatoid arthritis)

□⑬ Chronic hepatitis

□⑭ Allergy

□⑮ Others (disease name ____________)

Q4. Hot spring type

Please check the relevant box(es) to indicate which types of hot spring you have used.

□① Simple

□② Carbon dioxide

□③ Carbonate

□④ Chloride

□⑤ Sulfate

□⑥ Ferruginous

□⑦ Lodine-containing

□⑧ Acidic

□⑨ Radioactive

□⑩ Others (hot spring name ____________)

Q5. Time of hot spring bathing

Start time:

□① 7 P.M.

□② 8 P.M.

□③ 9 P.M.

□④ 10 P.M.

□⑤ 11 P.M. ______min

End time:

□① 7 P.M.

□② 8 P.M.

□③ 9 P.M.

□④ 10 P.M.

□⑤ 11 P.M. ______min

Q6. Blood pressure before and after hot spring bathing

Before: _____/_____mmHg

After: _____/_____mmHg

Please write here any comments you have about this questionnaire:

_________________________________________________________
