# Supplementary material for: Night-time hot spring bathing is associated with improved blood pressure control: A mobile application and paper questionnaire study
Source: PLoS One. 2024 Nov 1;19(11):e0299023. doi: 10.1371/journal.pone.0299023 (PMC11530088; doi:10.1371/journal.pone.0299023)
Supplement: Attachment — Submitted filename: PLOS one case on hot spring and HTN .pdf [file pone.0299023.s003.pdf]

PLOS ONE

Night-time hot spring bathing is associated with improved blood pressure control: A mobile application and paper questionnaire study

--Manuscript Draft--

**Manuscript Number:**	PONE-D-23-34165
**Article Type:**	Research Article
**Full Title:**	Night-time hot spring bathing is associated with improved blood pressure control: A mobile application and paper questionnaire study
**Short Title:**	Questionnaires on night-time hot springs
**Corresponding Author:**	SATOSHI YAMASAKI<br>Kyushu University Beppu Hospital: Kyushu Daigaku Byoin Beppu Byoin<br>Beppu, Oita JAPAN
**Keywords:**	questionnaire; mobile application; paper; hot spring bathing; night-time; blood pressure; Hypertension
**Abstract:**	Hot spring bathing is practiced helping manage hypertension. However, the details of the relationship between hot spring bathing and hypertension remain unknown. Older people are thought to be less adept at using digital devices, such as mobile applications, than younger people. Whether mobile application questionnaires, which have been increasing in recent years, can be used by older people is unclear. To address the knowledge gap regarding the management of older patients with hypertension, we prospectively evaluated mobile application and paper questionnaires regarding night-time hot spring bathing in respondents who had a choice of which to use. Changes in blood pressure because of hot spring bathing were evaluated. To investigate the effects of night-time hot spring bathing on blood pressure in adults, 1116 volunteers at 14 institutions in Beppu completed the study, including 562 in the mobile application questionnaire group and 556 in the paper questionnaire group. A total of 474 of 477 (99.3%) respondents aged ≥65 years used paper questionnaires. There was a significantly lower drop in systolic and diastolic blood pressure after using hot springs in respondents aged ≥65 years than in respondents aged <65 years ($p<0.001$). An age ≥65 years, hypertension with medication, arrhythmia, depression, and using a chloride hot spring were independently and significantly associated with a lower drop in systolic and diastolic blood pressure after night-time hot spring bathing ($p<0.001$). Night-time hot spring bathing is significantly associated with a reduced blood pressure in older adults ($p<0.001$). Extending our research further by examining how psychosocial factors in respondents aged ≥65 years may influence the preferences between mobile and paper questionnaires may be beneficial, and further investigation is warranted.
**Order of Authors:**	SATOSHI YAMASAKI
	Yusuke Kashiwado
	Toyoki Maeda
	Takahiko Horiuchi
**Additional Information:**	
**Question**	**Response**
**Financial Disclosure**<br><br>Enter a financial disclosure statement that describes the sources of funding for the work included in this submission. Review the submission guidelines for detailed requirements. View published research articles from *PLOS ONE* for specific	The author(s) received no specific funding for this work.

Powered by Editorial Manager® and ProduXion Manager® from Aries Systems Corporation

examples.<br><br>This statement is required for submission and **will appear in the published article** if the submission is accepted. Please make sure it is accurate.<br><br>**Funded studies**<br>Enter a statement with the following details:<br>• Initials of the authors who received each award<br>• Grant numbers awarded to each author<br>• The full name of each funder<br>• URL of each funder website<br>• Did the sponsors or funders play any role in the study design, data collection and analysis, decision to publish, or preparation of the manuscript?<br><br>Did you receive funding for this work?	
**Competing Interests**<br><br>Use the instructions below to enter a competing interest statement for this submission. On behalf of all authors, disclose any competing interests that could be perceived to bias this work—acknowledging all financial support and any other relevant financial or non-financial competing interests.<br><br>This statement is required for submission and **will appear in the published article** if the submission is accepted. Please make sure it is accurate and that any funding sources listed in your Funding Information later in the submission form are also declared in your Financial Disclosure statement.<br><br>View published research articles from *PLOS ONE* for specific examples.	NO authors have competing interests

Powered by Editorial Manager® and ProduXion Manager® from Aries Systems Corporation

**NO authors have competing interests**<br><br>Enter: *The authors have declared that no competing interests exist.*<br><br>**Authors with competing interests**<br><br>Enter competing interest details beginning with this statement:<br><br>*I have read the journal's policy and the authors of this manuscript have the following competing interests: [insert competing interests here]*<br><br>* typeset	
**Ethics Statement**<br><br>Enter an ethics statement for this submission. This statement is required if the study involved:<br><br>• Human participants<br>• Human specimens or tissue<br>• Vertebrate animals or cephalopods<br>• Vertebrate embryos or tissues<br>• Field research<br><br>Write "N/A" if the submission does not require an ethics statement.<br><br>General guidance is provided below. Consult the submission guidelines for detailed instructions. **Make sure that all information entered here is included in the Methods section of the manuscript.**	This was a prospective study with no experimental interventions.

Powered by Editorial Manager® and ProduXion Manager® from Aries Systems Corporation

**Format for specific study types**<br><br>**Human Subject Research (involving human participants and/or tissue)**<br>• Give the name of the institutional review board or ethics committee that approved the study<br>• Include the approval number and/or a statement indicating approval of this research<br>• Indicate the form of consent obtained (written/oral) or the reason that consent was not obtained (e.g. the data were analyzed anonymously)<br><br>**Animal Research (involving vertebrate animals, embryos or tissues)**<br>• Provide the name of the Institutional Animal Care and Use Committee (IACUC) or other relevant ethics board that reviewed the study protocol, and indicate whether they approved this research or granted a formal waiver of ethical approval<br>• Include an approval number if one was obtained<br>• If the study involved *non-human primates*, add *additional details* about animal welfare and steps taken to ameliorate suffering<br>• If anesthesia, euthanasia, or any kind of animal sacrifice is part of the study, include briefly which substances and/or methods were applied<br><br>**Field Research**<br><br>Include the following details if this study involves the collection of plant, animal, or other materials from a natural setting:<br>• Field permit number<br>• Name of the institution or relevant body that granted permission	
**Data Availability**<br><br>Authors are required to make all data underlying the findings described fully available, without restriction, and from the time of publication. PLOS allows rare exceptions to address legal and ethical concerns. See the PLOS Data Policy and FAQ for detailed information.	No - some restrictions will apply

Powered by Editorial Manager® and ProduXion Manager® from Aries Systems Corporation

A Data Availability Statement describing where the data can be found is required at submission. Your answers to this question constitute the Data Availability Statement and **will be published in the article**, if accepted.<br><br>**Important:** Stating 'data available on request from the author' is not sufficient. If your data are only available upon request, select 'No' for the first question and explain your exceptional situation in the text box.<br><br>Do the authors confirm that all data underlying the findings described in their manuscript are fully available without restriction?	
**Describe where the data may be found in full sentences. If you are copying our sample text, replace any instances of XXX with the appropriate details.**<br><br>• If the data are **held or will be held in a public repository**, include URLs, accession numbers or DOIs. If this information will only be available after acceptance, indicate this by ticking the box below. For example: *All XXX files are available from the XXX database (accession number(s) XXX, XXX.*).<br>• If the data are all contained **within the manuscript and/or Supporting Information files**, enter the following: *All relevant data are within the manuscript and its Supporting Information files.*<br>• If neither of these applies but you are able to provide **details of access elsewhere**, with or without limitations, please do so. For example:<br><br>*Data cannot be shared publicly because of [XXX]. Data are available from the XXX Institutional Data Access / Ethics Committee (contact via XXX) for researchers who meet the criteria for access to confidential data.*<br><br>*The data underlying the results presented in the study are available from (include the name of the third party*	The datasets generated and/or analyzed during the current study are not publicly available owing to privacy and confidentiality restrictions pertaining to personal health information. However, the dataset creation plan is available from the corresponding author on reasonable request.

and contact information or URL).<br>• This text is appropriate if the data are owned by a third party and authors do not have permission to share the data.<br><br>* typeset	
Additional data availability information:	

Powered by Editorial Manager® and ProduXion Manager® from Aries Systems Corporation

Manuscript

Click here to access/download;Manuscript;Yamasaki et al. app_Text 20231018.docx

Research Article

Night-time hot spring bathing is associated with improved blood pressure control: A mobile application and paper questionnaire study

Satoshi Yamasaki[1, 2*], Yusuke Kashiwado[1], Toyoki Maeda[1], Takahiko Horiuchi[1]

[1]Department of Internal Medicine, Kyushu University Beppu Hospital, Beppu, Japan

[2]Department of Hematology and Clinical Research Institute, National Hospital Organization Kyushu Medical Center, Fukuoka, Japan

Running title: Questionnaires on night-time hot springs

[*]Corresponding author

E-mail: yamas009@gmail.com (SY)

// Abstract

Hot spring bathing is practiced helping manage hypertension. However, the details of the relationship between hot spring bathing and hypertension remain unknown. Older people are thought to be less adept at using digital devices, such as mobile applications, than younger people. Whether mobile application questionnaires, which have been increasing in recent years, can be used by older people is unclear. To address the knowledge gap regarding the management of older patients with hypertension, we prospectively evaluated mobile application and paper questionnaires regarding night-time hot spring bathing in respondents who had a choice of which to use. Changes in blood pressure because of hot spring bathing were evaluated. To investigate the effects of night-time hot spring bathing on blood pressure in adults, 1116 volunteers at 14 institutions in Beppu completed the study, including 562 in the mobile application questionnaire group and 556 in the paper questionnaire group. A total of 474 of 477 (99.3%) respondents aged ≥65 years used paper questionnaires. There was a significantly lower drop in systolic and diastolic blood pressure after using hot springs in respondents aged ≥65 years than in respondents aged <65 years (p<0.001). An age ≥65 years, hypertension with medication, arrhythmia, depression, and using a chloride hot spring were independently and significantly associated with a lower drop in systolic

and diastolic blood pressure after night-time hot spring bathing (*p*<0.001). Night-time hot spring bathing is significantly associated with a reduced blood pressure in older adults (*p*<0.001). Extending our research further by examining how psychosocial factors in respondents aged ≥65 years may influence the preferences between mobile and paper questionnaires may be beneficial, and further investigation is warranted.

Keywords: questionnaire; mobile application; paper; hot spring bathing; night-time; blood pressure; hypertension

Introduction

The prevalence of hypertension is high in the world [1]. The control of blood pressure to a systolic pressure of <130 mmHg and a diastolic pressure of <80 mmHg after taking antihypertensive drug therapy was achieved by 54%, 50%, 46%, and 33% of patients aged 20–54 years, 55–64 years, 65–74 years, and ≥75 years, respectively [2]. The treatment of hypertension should involve lifestyle modifications, such as dietary salt restriction [3], weight loss [4], better diet [5], exercise [6], limited alcohol intake [1], reduced smoking habits [7], reduced consumption of coffee and tea [8], more sleep

[9], and hot spring bathing in older patients [10]. However, the details of the relationship between hot spring bathing and hypertension remain unknown.

In Japan, there is a long history of habitual daily bathing, which involves hot bathing rather than showering. In hot springs areas, including Beppu, which contains the world highest concentration of hot spring sources and has 9 of the 10 hot spring types, people appear to habitually prefer bathing in hot springs. Hot spring bathing, which has balneotherapeutic effects according to the hot spring type as reported by the Japanese Ministry of the Environment [11], is more effective for raising core body temperature than hot bathing.

Medical questionnaires are widely administered in numerous medical fields, including those regarding hypertension [12], and are used by many specialists. Using paper questionnaires is important to assess the effect of hot spring bathing on health-related issues, but the area for questionnaire use has been limited to a small area, including a city [13]. The benefits of mobile application questionnaires include the ease of use, immediate electronic storage of results and automatic scoring, avoidance of secondary data entry errors, and easier follow-up of records with time [14]. Additionally, there has been positive feedback on mobile application questionnaires,

which were applied for informed consent procedures, to assess the quality of life [15], medical education [16], interventions, diagnostics, and questionnaire completion [17].

To address the knowledge gap regarding the management of older patients with hypertension, we prospectively evaluated the use of mobile application and paper questionnaires regarding hot spring bathing and evaluated a drop in blood pressure because of hot spring bathing. The primary aim of this study was to allow adults aged ≥65 years to choose what type of questionnaire to use. We also measured variables that could affect blood pressure, such as demographics, medical history, and concomitant medications. We performed univariate and multivariate regression analyses to examine the correlations between these variables. We identified which variables were dependent variables on the basis of our prespecified hypothesis of an inverse correlation between night-time hot spring bathing and blood pressure, as reported in a previous cross-sectional study [10].

Materials and Methods

Study population

In this study, we developed a mobile application questionnaire from Neo Marketing Inc., Tokyo, Japan, and a paper (S1 Table) questionnaire. When completing electronic

questionnaires on a mobile phone, an interface from Neo Marketing Inc. is sent to the mobile application questionnaires, while another interface from the mobile phone is sent to data storage in Neo Marketing Inc. This system enables adults to answer mobile application questionnaires directly on their mobile phone while enabling questionnaire researchers to quickly obtain all necessary questionnaires through the mobile application and analysis.

The study population included 1116 adults aged $\geq$18 years who were using night-time hot springs at 14 institutes (6 members of the Beppu Tourist Hotel Association, 7 hot springs organized by Beppu City, and our institute) in Beppu. We included 434 men and 684 women aged 22–94 years who met the inclusion criteria, provided consent, and were enrolled in this study between 26 June 2023 and 30 September 2023. The inclusion criteria were men and women aged at least 18 years who could read and who used night-time hot springs. In this study, the primary end point was that 80% of the respondents were able to complete the required questions. Assuming that 85% of respondents could complete the answer, the α error was 0.05, the power was 0.80, and n=365 subjects were required. Therefore, the number of respondents was 400 for each form and application.

Initially, we used a questionnaire using a mobile application, but the respondents could also use a paper questionnaire if they preferred. This selection between mobile and paper, which was based on sex, age, and a history of disease, was used for further analysis of the data. All of the data were collected and double-checked for accuracy by two separate research assistants. We examined the following variables: sex; age; hypertension with or without medication; and the lifetime disease history, including arrhythmia, stroke, gout, diabetes mellitus, hyperlipidemia, renal disease, and chronic hepatitis, which are associated with a lower prevalence of hypertension [10]. Informed consent for study participation was obtained by providing the respondents with information on mobile or paper questionnaires. The study was performed in accordance with the institutional guidelines and the principles of the Declaration of Helsinki. The protocol was approved by the institutional review board of Kyushu University Hospital, Japan (No. 22346-00).

Statistical methods

The prespecified primary analyses of adult favorability of a mobile phone or a paper form of the questionnaire were based on the chi-square test to determine whether the proportion of adults who considered a questionnaire by mobile phone to be the same,

better, or much better than paper was significantly >50%. Similarly, adult preferences were analyzed in stratified groups on the basis of sex, age (≥65 vs. <65 years old), a history of diseases, and type of hot spring as defined by the Japan Spa Association (https://www.spa.or.jp/en/spring-quality/; e.g., a "chloride spring" may include sodium chloride, calcium chloride, and magnesium chloride springs, depending on the main ingredients of the cation). We analyzed the frequencies and descriptive statistics of respondents' variables. We excluded respondents if they had missing data for at least one of the covariates. Intergroup differences in categorical variables are expressed as the number and percentage. The chi-square test was used to examine the relationships between categorical variables.

Univariate and multivariate regression analyses were conducted to determine the associations between the variables and a drop in systolic or diastolic blood pressure after night-time hot spring bathing. Covariates that were significant at $p<0.05$ in the univariate regression analysis were included in the multivariate regression analysis. We prospectively calculated the coefficient and 95% confidence intervals of the effect of a change in systolic and diastolic blood pressure before and after using night-time hot springs using the multivariate regression model. A p value <0.05 was considered statistically significant. All analyses were conducted using EZR (Saitama Medical

Center, Saitama, Japan; http://www.jichi.ac.jp/saitama-sct/SaitamaHP.files/statmedEN.html) [18], which is a graphical user interface for R version 2.13.0 (www.r-project.org), and a modified version of R Commander version 1.6-3 designed to add statistical functions.

Results

The baseline characteristics of the respondents who used night-time hot springs are shown in Table 1. The median (range) age of the respondents who used night-time hot springs in the mobile application and paper questionnaire groups was 48 (22–86) and 79 (60–94) years, respectively. The respondents in the mobile application questionnaire group were younger than the those in the paper questionnaire group (p<0.001).

Table 1. Characteristics of the survey respondents.

Characteristic		Mobile n = 562	Paper n = 556	*p*
**Gender, n (%)**	**Male**	**158 (28.1)**	**276 (49.6)**	**<0.001**
	**Female**	**404 (71.9)**	**280 (50.4)**	
**Median age (range), years**		**48 (22-86)**	**79 (60-94)**	**<0.001**
**≥65 years old, n (%)**		**3 (0.5)**	**474 (85.3)**	**<0.001**
**Disease history, n (%)**				
**Hypertension with medication**		**288 (51.2)**	**452 (81.3)**	**<0.001**
**Hypertension without medication**		**271 (48.2)**	**84 (15.1)**	**<0.001**
**Cancer**		**2 (0.4)**	**102 (18.3)**	**<0.001**
**Acute myocardial infarction and angina**		**3 (0.5)**	**105 (18.9)**	**<0.001**
**Arrhythmia**		**3 (0.5)**	**94 (16.9)**	**<0.001**
**Stroke**		**0**	**102 (18.3)**	**-**
**Gout**		**5 (0.9)**	**51 (9.2)**	**<0.001**
**Diabetes mellitus**		**17 (3.0)**	**254 (45.7)**	**<0.001**
**Hyperlipidemia**		**112 (19.9)**	**220 (39.6)**	**<0.001**

**Renal disease**	**3 (0.5)**	**153 (27.5)**	**<0.001**
**Depression**	**391 (69.6)**	**187 (33.6)**	**<0.001**
**Collagen disease**	**1 (0.2)**	**0**	**-**
**Hot spring-bathing, n (%)**			
**Hot spring type**			
**Simple**	**98 (17.4)**	**72 (12.9)**	**<0.001**
**Chloride**	**464 (82.6)**	**484 (87.1)**	**0.044**
**Time**			
**19:00 to 20:00**	**307 (54.6)**	**457 (82.2)**	**<0.001**
**20:00 to 21:00**	**215 (38.3)**	**53 (9.5)**	
**21:00 to 22:00**	**34 (6.0)**	**46 (8.3)**	
**22:00 or 23:00**	**6 (1.1)**	**0**	
**Median (range) BP before bathing, mmHg**			
**Systolic BP**	**156 (150-159)**	**154 (140-159)**	**<0.001**
**Diastolic BP**	**93 (87-94)**	**94 (75-98)**	**<0.001**
**Median (range) BP after bathing, mmHg**			
**Systolic BP**	**145 (112-147)**	**126 (112-149)**	**<0.001**
**Diastolic BP**	**85 (65-89)**	**75 (64-88)**	**<0.001**
**Median (range) drip of BP after bathing, mmHg**			
**Systolic BP**	**11 (9-44)**	**28 (3-47)**	**<0.001**
**Diastolic BP**	**8 (1-26)**	**17 (3-31)**	**<0.001**

The *p* values were obtained using the chi-square test.
BP, blood pressure.

To evaluate the association between age (≥65 vs. <65 years old) and a drop in systolic or diastolic blood pressure, we compared the drop in systolic (median: 27 vs. 11 mmHg, range: 3–47 vs. 7–36 mmHg, respectively) and diastolic (median: 17 vs. 8 mmHg, range: 1–31 vs. 1–26 mmHg, respectively) blood pressure after using a night-time hot spring (Fig 1a and b, respectively). The drop in systolic and diastolic blood pressure was significantly lower in respondents aged ≥65 years than in respondents aged <65 years (p<0.001).

Fig 1a. Drop in systolic blood pressure after night-time hot spring bathing.

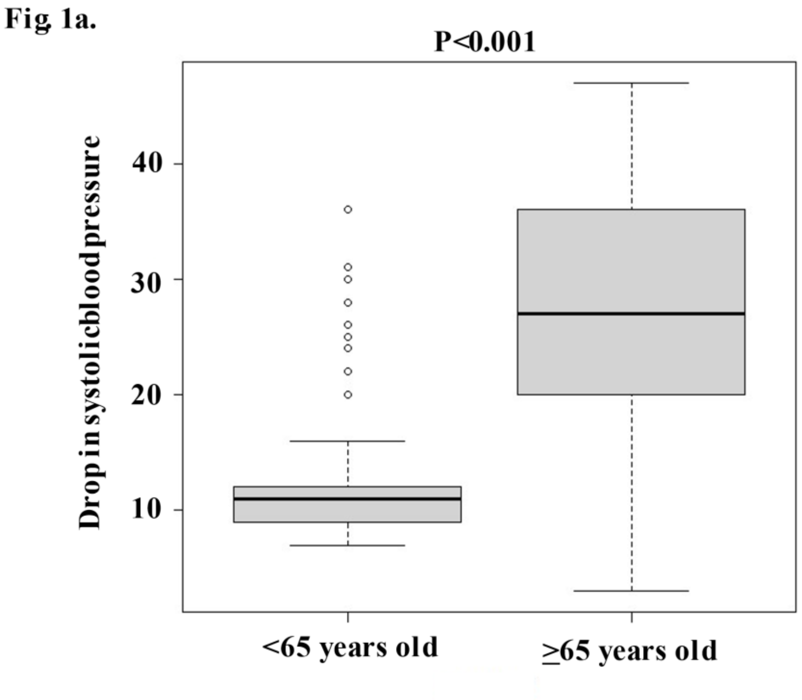

Fig 1b. Drop in diastolic blood pressure after night-time hot spring bathing.

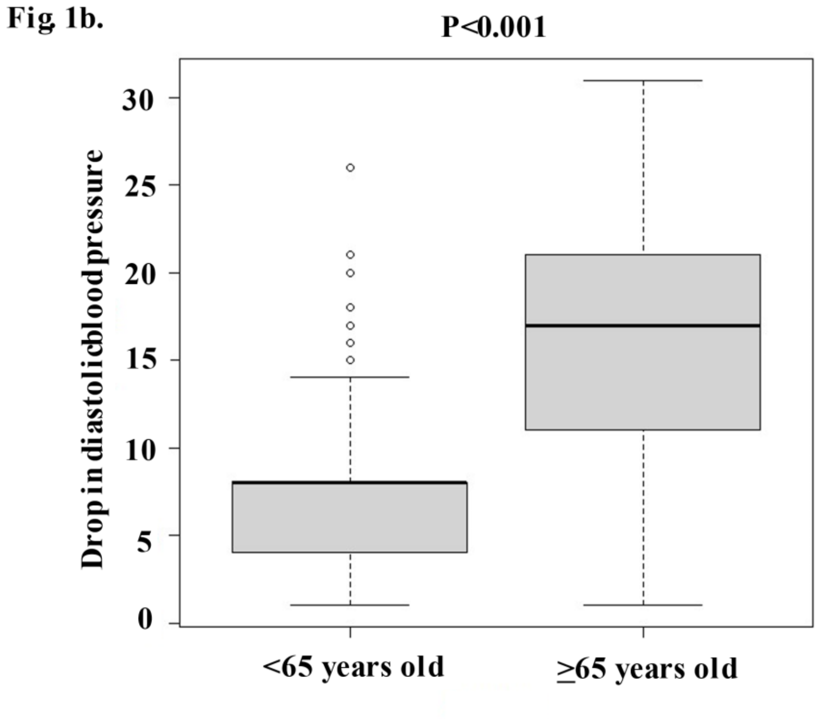

In the multivariate regression analysis, male sex (p<0.001), age ≥65 years (p<0.001), a disease history including hypertension with medication (p<0.001), hypertension without medication (p=0.007), arrhythmia (p<0.001), gout (p<0.001), and depression (p<0.001), and using a chloride hot spring (p<0.001) were significantly associated with a drop in systolic blood pressure after night-time hot spring bathing (Table 2). An age ≥65 years (p<0.001), a disease history including hypertension with medication (p<0.001), arrhythmia (p<0.001), and depression (p<0.001), and using a chloride hot spring ($p < 0.001$) were significantly associated with ac drop in diastolic blood pressure after night-time hot spring bathing (Table 3).

Table 2. Univariable and multivariable regression analyses of variables affecting the drop in systolic blood pressure after night-time hot spring bathing.

		Univariable				Multivariable			
Parameter	***Variable***	***Coefficient***	**95% CI**	***s.e.***	***p***	***Coefficient***	**95% CI**	***s.e.***	***p***
Gender	**Male vs. Female**	**2.113**	**0.975-3.251**	**0.580**	**<0.001**	**2.098**	**0.960- 3.237**	**0.580**	**<0.001**
Age	**≥65 years old vs.<65 years old**	**12.697**	**11.465-13.929**	**0.627**	**<0.001**	**13.103**	**11.960- 14.245**	**0.582**	**<0.001**
Disease history	**Hypertension with medication**	**6.215**	**3.117-9.313**	**1.578**	**<0.001**	**6.060**	**2.966- 9.154**	**1.577**	**<0.001**
	**Hypertension without medication**	**4.381**	**1.214-7.547**	**1.613**	**0.006**	**4.319**	**1.151- 7.487**	**1.614**	**0.007**
	**Cancer**	**-0.342**	**-2.137-1.452**	**0.914**	**0.708**				
	**Acute myocardial infarction and angina**	**-12.041**	**-15.107- -8.976**	**1.562**	**<0.001**	**-10.722**	**-13.412- -8.033**	**1.370**	**<0.001**
	**Arrhythmia**	**11.861**	**9.379-14.343**	**1.265**	**<0.001**	**10.961**	**8.678-13.244**	**1.163**	**<0.001**
	**Gout**	**6.595**	**3.135-10.055**	**1.763**	**<0.001**	**5.577**	**2.384-8.770**	**1.627**	**<0.001**
	**Diabetes mellitus**	**-5.008**	**-6.394- -3.622**	**0.706**	**<0.001**	**-5.210**	**-6.566- -3.854**	**0.691**	**<0.001**
	**Hyperlipidemia**	**-1.746**	**-2.891- -0.600**	**0.583**	**0.002**	**-1.537**	**-2.597- -0.477**	**0.540**	**0.004**
	**Renal disease**	**-1.457**	**-3.065-0.150**	**0.819**	**0.075**				
	**Depression**	**3.697**	**2.562-4.832**	**0.578**	**<0.001**	**3.638**	**2.518-4.758**	**0.570**	**<0.001**
Hot spring type	**Chloride vs. Simple**	**3.760**	**2.546-4.974**	**0.618**	**<0.001**	**3.872**	**2.663-5.080**	**0.615**	**<0.001**

s.e., standard error; CI, confidence interval.
†Univariable or multivariable competing event statistics analyzed using a regression model were applied to a decrease in systolic blood pressure after night-time hot spring bathing. Covariates significant at *p*<0.05 in the univariate analysis were included in the multivariate analysis.

Table 3. Univariable and multivariable regression analyses of variables affecting the drop in diastolic blood pressure after night-time hot spring bathing.

		Univariable				Multivariable			
Parameter	***Variable***	***Coefficient***	**95% CI**	***s.e.***	***p***	***Coefficient***	**95% CI**	***s.e.***	***p***
Gender	**Male vs. Female**	**-0.185**	**-0.803-0.432**	**0.315**	**0.556**				
Age	**≥65 years old vs.<65 years old**	**5.366**	**4.697-6.035**	**0.341**	**<0.001**	**5.377**	**4.713-6.042**	**0.338**	**<0.001**
Disease history	**Hypertension with medication**	**2.585**	**0.902-4.268**	**0.857**	**0.002**	**1.455**	**0.912-1.997**	**0.276**	**<0.001**
	**Hypertension without medication**	**1.147**	**-0.572-2.867**	**0.876**	**0.190**				
	**Cancer**	**-2.216**	**-3.191- -1.240**	**0.496**	**<0.001**	**-1.971**	**-2.908- -1.035**	**0.477**	**<0.001**
	**Acute myocardial infarction and angina**	**-7.351**	**-9.016- -5.685**	**0.848**	**<0.001**	**-6.361**	**-7.499- -5.223**	**0.579**	**<0.001**
	**Arrhythmia**	**10.021**	**8.672-11.369**	**0.687**	**<0.001**	**9.474**	**8.322-10.627**	**0.587**	**<0.001**
	**Gout**	**1.716**	**-0.1633-3.595**	**0.957**	**0.073**				
	**Diabetes mellitus**	**-6.876**	**-7.629- -6.124**	**0.383**	**<0.001**	**-7.089**	**-7.809- -6.368**	**0.367**	**<0.001**
	**Hyperlipidemia**	**-2.861**	**-3.483- -2.239**	**0.317**	**<0.001**	**-2.791**	**-3.396- -2.186**	**0.308**	**<0.001**
	**Renal disease**	**-3.422**	**-4.295- -2.548**	**0.445**	**<0.001**	**-3.206**	**-4.046- -2.365**	**0.428**	**<0.001**
	**Depression**	**1.478**	**0.862-2.095**	**0.314**	**<0.001**	**1.464**	**0.941-1.987**	**0.266**	**<0.001**
Hot spring type	**Chloride vs. Simple**	**2.016**	**1.357-2.676**	**0.336**	**<0.001**	**2.087**	**1.431-2.743**	**0.334**	**<0.001**

s.e., standard error; CI, confidence interval.
†Univariable or multivariable competing event statistics analyzed using a regression model were applied to a decrease in diastolic blood pressure after night-time hot spring bathing. Covariates significant at *p*<0.05 in the univariate analysis were included in the multivariate analysis.

In the multivariate regression analysis, a disease history, including acute myocardial infarction and angina (*p*<0.001), diabetes mellitus (*p* 0.001), and hyperlipidemia (*p*=0.004), was inversely associated with a drop in systolic blood pressure after night-time hot spring bathing (Table 2). A disease history, including cancer (*p*<0.001), acute

myocardial infarction and angina ($p<0.001$), diabetes mellitus ($p<0.001$), hyperlipidemia ($p<0.001$), and renal disease ($p<0.001$), was inversely associated with a drop in diastolic blood pressure after night-time hot spring bathing (Table 3).

Discussion

This study showed that 99.3% of respondents aged ≥65 years used paper questionnaires. There was a significantly lower drop in systolic and diastolic blood pressure after using hot springs in respondents aged ≥65 years than in respondents aged <65 years. An age ≥65 years, a disease history including hypertension with medication, arrhythmia, and depression, and using a chloride hot spring were independently and significantly associated with a drop in systolic and diastolic blood pressure after night-time hot spring bathing.

In this prospective study, adults aged ≥65 years preferred to use paper questionnaires over mobile application questionnaires. Studies on an electronic questionnaire using tablet computers reported no difficulty in the majority of patients, except older age, those with less than a high school education, and the presence of comorbid medical conditions [19, 20]. As in previous studies, mobile questionnaires were favored by younger patients with a higher education level [20]. Additionally,

patients who use a mobile device daily appear to be more likely to prefer mobile questionnaires than those who do not use a mobile device daily [20]. Extending our research to adults aged ≥65 years by examining how psychosocial factors, such as self-efficacy, as well as physical factors such as visual or memory impairment, may influence the preferences between mobile and paper questionnaires may be beneficial. We believe that the ease and feasibility of mobile questionnaires might help increase respondents' satisfaction and preference using mobile questionnaires over paper questionnaires.

To date, only one study has investigated the association between habitual night-time hot spring bathing and a lower prevalence of hypertension [10]. Traditional thermal therapy, including hot spring bathing, has proven useful for hypertension in previous studies [21, 22, 23]. The present study showed that night-time hot spring bathing resulted in a drop in systolic and diastolic blood pressure. The drop in systolic and diastolic blood pressure after night-time bathing was significantly better in respondents aged ≥65 years than in those aged <65 years. Tai *et al*. reported that Japanese hot water bathing, especially in the short time from the end of bathing to bedtime, was associated with lower night-time and sleep-time blood pressure and greater dipping in an older

adult population [24]. Therefore, night-time hot spring bathing might be more suitable for older adults than younger adults to reduce blood pressure.

The present study showed that hypertension with medication or arrhythmia was independently and significantly associated with a drop in systolic and diastolic blood pressure after night-time hot spring bathing. This finding indicates the need for specific intervention strategies aimed at older adults living with hypertension or arrhythmia, one of which could be night-time hot spring bathing. Systolic blood pressure is considered the major predictor of coronary disease in older patients [25]. An elevation in systolic blood pressure is primarily due to diminished arterial compliance, and isolated systolic hypertension may result from an increase in cardiac output due to anemia, aortic insufficiency, and arteriovenous fistula [26]. Although understanding the effect of hot spring bathing on decreasing the risk of coronary heart disease is important and requires further research, this might be particularly relevant to the decreased risk of coronary heart disease in older patients after hot spring bathing. In this study, we did not follow the baseline blood pressure because of the difficulty of data collection. To evaluate whether the anti-hypertensive effects of hot spring bathing are persistent, we are currently conducting a prospective, longitudinal study in hospitalized patients with hypertension.

In the multivariate regression analysis, we found that the depression was independently and significantly associated with a drop in systolic and diastolic blood pressure after night-time hot spring bathing. We found that night-time hot spring bathing, which can improve sleep disorders due to depression, might be associated with a drop in systolic and diastolic blood pressure in older patients. In a large-scale study of an older population, night-time hot spring bathing was associated with shorter sleep onset latency when bathing was scheduled 1–3 hours before bedtime, and with a higher distal–proximal skin temperature gradient if bathing occurred 30 minutes before bedtime [27]. Brief exposure to sauna bathing can result in benefits for <1 hour, such as reduced blood pressure and improved arterial stiffness. However, sauna bathing for ≥3 weeks with repeated frequency can upregulate certain enzymes and pathways, which results in greater stress tolerance, an enhanced cellular environment, and improved health [23]. Owing to la ack of physical activity and healthy nutrition in older patients with depression, practical interventions to prevent or improve hypertension, such as habitual night-time hot spring bathing, warrant additional attention.

Our study showed that hot spring bathing in a chloride spring bath was independently and significantly associated with a drop in the systolic and diastolic blood pressure after night-time hot spring bathing. Therefore, people with hypertension

may find engaging in a chloride spring bath beneficial. The effect of sodium chloride concentrations on physical and oxidative stability has been shown to be useful in cooking [28], and a clinical trial showed that sodium chloride was highly efficient in increasing core body temperature [29]. Additionally, a small Japanese study suggested that hot spring bathing in water containing sodium chloride maintained a higher rate of heat stability and was more efficient in elevating core body temperature than bathing in hot spring water without sodium chloride [29]. However, these findings should be confirmed in a prospective study with a control group, which is outside of the scope of our current study.

There are some limitations to this study. First, some selection bias was expected in this prospective study, which was performed at 14 institutes. In this study, bias was present owing to the following differences in data selection and other factors: a lack of data regarding respondents who engaged in hot spring bathing for the treatment of various diseases; respondents' income, which might be correlated with some vascular diseases or the frequency of hot spring bathing; respondents' lifestyle and diet, such as food consumption and obesity, sodium intake, drinking and smoking habits, consumption of coffee and tea, physical activity, and sleep; and respondents' time of hot spring bathing. To minimize the bias, we limited the inclusion criteria to patients with

data on sex, age, disease history, and use of night-time hot spring bathing. Second, no long-term data on blood pressure were available. Therefore, further studies are required to assess the details of the long-term outcomes of patients who undergo hot spring bathing. Third, patients with hypertension may have been overlooked or the changes in blood pressure underestimated because of antihypertensive drug therapy. Additionally, there were no data specific to hot spring bathing, such as the duration of immersion, frequency, temperature, time, and years of habitual hot spring bathing. Finally, there is a lack of generalizability of the findings of this study to other populations.

Conclusions

Even though there has been increasing administration of mobile questionnaires in numerous medical fields, we found that 99.3% respondents aged ≥65 years used paper questionnaires. Mobile questionnaires have the possibility of increasing clinical efficiency, improving physician–patient communication, and improving the patient's encounter overall. Therefore, the ease and feasibility of mobile questionnaires might help increase respondents' preference using these questionnaires. This study showed a significantly lower drop in systolic and diastolic blood pressure after using hot springs

in respondents aged ≥65 years than in respondents aged <65 years. Night-time hot spring bathing was significantly associated with reduced blood pressure in older adults. Further extending our research by examining how psychosocial factors may influence the preferences between mobile and paper questionnaires could be beneficial, and further investigation is warranted.

Questionnaire regarding night-time hot spring bathing and blood pressure

Q1. Sex

1. Male 2. Female

Q2. Age

_______ years old

Q3. Disease history

Please place a check next to any disease that you have experienced.

□① Hypertension (with medication)
□② Hypertension (without medication)
□③ Cancer
□④ Acute myocardial infarction and angina
□⑤ Arrythmia
□⑥ Stroke
□⑦ Gout
□⑧ Diabetes mellites
□⑨ Hyperlipidemia
□⑩ Renal disease
□⑪ Depression
□⑫ Collagen disease (e.g., rheumatoid arthritis)
□⑬ Chronic hepatitis
□⑭ Allergy

□⑮ Others (disease name ____________)

Q4. Hot spring type
Please place a check next to any type of hot spring that you have used.
□① Simple
□② Carbon dioxide
□③ Carbonate
□④ Chloride
□⑤ Sulfate
□⑥ Ferruginous
□⑦ Lodine-containing
□⑧ Acidic
□⑨ Radioactive
□⑩ Others (hot spring name ____________)

Q5. Time of hot spring bathing
Start time:
□① 7 P.M.
□② 8 P.M.
□③ 9 P.M.
□④ 10 P.M.
□⑤ 11 P.M. ______min

End time:
□① 7 P.M.
□② 8 P.M.
□③ 9 P.M.
□④ 10 P.M.
□⑤ 11 P.M. ______min

Q6. Blood pressure before and after hot spring bathing
Before: _____/_____mmHg
After: _____/_____mmHg

Please write any free comments that you may have regarding the questionnaire:
____

S1 Table.
English version of the Japanese questionnaire used in this study.

Data availability

We used data obtained from patients' medical records at Kyushu University Beppu Hospital, Beppu, Japan. The datasets generated and/or analyzed during the current study are not publicly available owing to privacy and confidentiality restrictions pertaining to personal health information. However, the dataset creation plan is available from the corresponding author on reasonable request.

Acknowledgments

We thank the respondents and clinical staff for their participation in the study. We appreciate Mr. Yasuhiro Nagano (Mayor of Beppu), the Tourist Division of Beppu City, and the Beppu Tourist Hotel Association, especially Hotel Sunbury Annex, Hanabeppu, and Amane Resort for enabling data collection. We thank Dr. Ellen Knapp, PhD, from Edanz (https://jp.edanz.com/ac) for editing a draft of this manuscript.

Author contributions

S. Y. designed the study, analyzed the data, and prepared the manuscript. S. Y., Y. K., T. M., and T. H. prepared and reviewed the manuscript.

References

1. Whelton PK, Carey RM, Aronow WS, Casey DE Jr., Collins KJ, Dennison Himmelfarb C, et al. 2017 ACC/AHA/AAPA/ABC/ACPM/AGS/APhA/ASH/ASPC/NMA/PCNA Guideline for the Prevention, Detection, Evaluation, and Management of High Blood Pressure in Adults: A Report of the American College of Cardiology/American Heart Association Task Force on Clinical Practice Guidelines. Hypertension. 2018; 71: e13–e115. DOI: 10.1161/HYP.0000000000000065 PMID: 29133356
2. Muntner P, Carey RM, Gidding S, Jones DW, Taler SJ, Wright JT Jr., et al. Potential US Population Impact of the 2017 ACC/AHA High Blood Pressure Guideline. Circulation. 2018; 137: 109–118. DOI: 10.1161/CIRCULATIONAHA.117.032582 PMID: 29133599
3. He FJ, Li J, Macgregor GA. Effect of longer term modest salt reduction on blood pressure: Cochrane systematic review and meta-analysis of randomised trials. British

Medical Journal. 2013; 346: f1325. DOI: 10.1136/bmj.f1325 PMID: 23558162

4. Tuck ML, Sowers J, Dornfeld L, Kledzik G, Maxwell M. The effect of weight reduction on blood pressure, plasma renin activity, and plasma aldosterone levels in obese patients. New England Journal of Medicine. 1981; 304: 930–933. DOI: 10.1056/NEJM198104163041602 PMID: 7010165
5. Forman JP, Stampfer MJ, Curhan GC. Diet and lifestyle risk factors associated with incident hypertension in women. JAMA. 2009; 302: 401–411. DOI: 10.1001/jama.2009.1060 PMID: 19622819
6. Carnethon MR, Evans NS, Church TS, Lewis CE, Schreiner PJ, Jacobs DR Jr., et al. Joint associations of physical activity and aerobic fitness on the development of incident hypertension: coronary artery risk development in young adults. Hypertension. 2010; 56: 49–55. doi: 10.1161/HYPERTENSIONAHA.109.147603. PMID: 20516395
7. Virdis A, Giannarelli C, Neves MF, Taddei S, Ghiadoni L. Cigarette smoking and hypertension. Curr Pharm Des. 2010; 16: 2518–2525. DOI: 10.2174/138161210792062920 PMID: 20550499
8. Teramoto M, Yamagishi K, Muraki I, Tamakoshi A, Iso H. Coffee and Green Tea Consumption and Cardiovascular Disease Mortality Among People With and Without

Hypertension. J Am Heart Assoc 2023; 12: e026477. DOI: 10.1161/JAHA.122.026477 PMID: 36542728

9. Carnethon MR, Johnson DA. Sleep and Resistant Hypertension. Curr Hypertens Rep. 2019; 21: 34. DOI: 10.1007/s11906-019-0941-z PMID: 30953264

10. Yamasaki S, Tokunou T, Maeda T, Horiuchi T. Hot spring bathing is associated with a lower prevalence of hypertension among Japanese older adults: a cross-sectional study in Beppu. Sci Rep. 2022; 12: 19462. DOI: 10.1038/s41598-022-24062-3 PMID: 36376349

11. https://www.env.go.jp/content/900492871.pdf#search, Japanese Ministry of Environment. 'The ABCs for the Safe and Secure Use of Onsen'.

12. Buis LR, Roberson DN, Kadri R, Rockey NG, Plegue MA, Danak SU, et al. Understanding the Feasibility, Acceptability, and Efficacy of a Clinical Pharmacist-led Mobile Approach (BPTrack) to Hypertension Management: Mixed Methods Pilot Study. J Med Internet Res. 2020; 22: e19882. doi: 10.2196/19882. PMID: 32780026

13. Maeda T, Mimori K, Suzuki S, Horiuchi T, Makino N. Preventive and promotive effects of habitual hot spa-bathing on the elderly in Japan. Sci Rep. 2018; 8: 133. DOI: 10.1038/s41598-017-18488-3 PMID: 29317745

14. Fritz F, Balhorn S, Riek M, Breil B, Dugas M. Qualitative and quantitative evaluation

of EHR-integrated mobile patient questionnaires regarding usability and cost-efficiency. Int J Med Inform. 2012; 81: 303–313. DOI: 10.1016/j.ijmedinf.2011.12.008 PMID: 22236957

15. Aiello EJ, Taplin S, Reid R., Hobbs M, Seger D, Kamel H, et al. In a randomized controlled trial, patients preferred electronic data collection of breast cancer risk-factor information in a mammography setting. J Clin Epidemiol. 2006; 59: 77–81. DOI: 10.1016/j.jclinepi.2005.07.007 PMID: 16360564

16. George P, Dumenco L, Doyle R, Dollase R. Incorporating iPads into a preclinical curriculum: a pilot study. Med Teach. 2013; 35: 226–230. DOI: 10.3109/0142159X.2012.735384 PMID: 23425119

17. Dorr M, Lesmes LA, Lu ZL, Bex PJ. Rapid and reliable assessment of the contrast sensitivity function on an iPad. Invest Ophthalmol Vis Sci. 2013; 54: 7266–7273. DOI: 10.1167/iovs.13-11743 PMID: 24114545

18. Kanda Y. Investigation of the freely available easy-to-use software 'EZR' for medical statistics. Bone Marrow Transplantation. 2013; 48: 452–458. DOI: 10.1038/bmt.2012.244 PMID: 23208313

19. Velikova G, Wright EP, Smith AB, Cull A, Gould A, Forman D, et al. Automated collection of quality-of-life data: a comparison of paper and computer touch-screen

questionnaires. J Clin Oncol. 1999; 17: 998–1007. DOI: 10.1200/JCO.1999.17.3.998 PMID: 10071295

20. Hess R, Santucci A, McTigue K, Fischer G, Kapoor W. Patient difficulty using tablet computers to screen in primary care. J Gen Intern Med. 2008; 23, 476–480. DOI: 10.1007/s11606-007-0500-1 PMID: 18373148

21. Kataoka T, Aoyama Y, Sakoda A, Nakagawa S, Yamaoka K. Basic study on biochemical mechanism of thoron and thermal therapy. Physiological Chemistry and Physics and Medical NMR. 2006; 38: 85–92. PMID: 18472469

22. Chen Z, Yang J, Zhu L, Zhang A, Wang Z, Liu P, et al. Classification of typical hot springs and their relationship with health in Guizhou, China. Evironmental Geochemistry and Health. 2021; 43: 1287–1304. DOI: 10.1007/s10653-020-00724-x PMID: 32974884

23. Henderson KN, Killen LG, O'Neal EK, Waldman HS. The Cardiometabolic Health Benefits of Sauna Exposure in Individuals with High-Stress Occupations. A Mechanistic Review. International Journal of Environmental Research and Public Health. 2021; 18: 1105. DOI: 10.3390/ijerph18031105 PMID: 33513711

24. Tai Y, Saeki K, Yamagami Y, Yoshimoto K, Kurumatani N, Nishio K, et al. Association between timing of hot water bathing before bedtime and night-/sleep-

time blood pressure and dipping in the elderly: a longitudinal analysis for repeated measurements in home settings. Chronobiology International. 2019; 36: 1714–1722. DOI: 10.1080/07420528.2019.1675685 PMID: 31610699

25. Franklin SS, Larson MG, Khan SA, Wong ND, Leip EP, Kannel WB, et al. Does the relation of blood pressure to coronary heart disease risk change with aging? The Framingham Heart Study. Circulation. 2001; 103: 1245–1249. DOI: 10.1161/01.cir.103.9.1245 PMID: 11238268

26. Chobanian AV. Clinical practice. Isolated systolic hypertension in the elderly. New England Journal of Medicine. 2007; 357: 789–796. DOI: 10.1056/NEJMcp071137 PMID: 17715411

27. Tai Y, Obayashi K, Yamagami Y, Yoshimoto K, Kurumatani N, Nishio K, et al. Hot-water bathing before bedtime and shorter sleep onset latency are accompanied by a higher distal-proximal skin temperature gradient in older adults. Journal of Clinical Sleep Medicine. 2021; 17: 1257–1266. DOI: 10.5664/jcsm.9180 PMID: 33645499

28. Min B, Cordray JC, Ahn DU. Effect of NaCl, myoglobin, Fe(II), and Fe(III) on lipid oxidation of raw and cooked chicken breast and beef loin. J Agric Food Chem. 2010; 58: 600–605. DOI: 10.1021/jf9029404 PMID: 19904983

29. Maeda T, Kudo Y, Horiuchi T, Makino N. Clinical and anti-aging effect of mud-bathing therapy for patients with fibromyalgia. Mol Cell Biochem. 2018; 444: 87–92. DOI: 10.1007/s11010-017-3233-4 PMID: 29214470
